# Thirty-second Joule-heating synthesis of Cu-intercalated Mo_6_S_8_ Chevrel phases with pH-symmetric hydrogen evolution behavior

**DOI:** 10.1039/d6ra05873f

**Published:** 2026-10-19

**Authors:** Zongchen Jiang, Wenqi Li, Zhenyu Chen, Shengnan Zhang, Botao Shao, Fan Yang, Ping Hu, Chengshan Li, Pingxiang Zhang

**Affiliations:** a Superconducting Materials Research Center, Northwest Institute for Non-ferrous Metal Research Xi'an 710016 China zychen@c-nin.com snzhang@c-nin.com; b School of Materials Science and Engineering, Northeastern University Shenyang 110004 China; c School of Metallurgy Engineering, Xi'an International Science and Technology Cooperation Base for Manufacturing of Special Powder and Powder Metallurgy, Xi'an University of Architecture and Technology Xi'an 710055 China yangfan1990@xauat.edu.cn huping@xauat.edu.cn

## Abstract

Chevrel-phase compounds are structurally robust transition-metal chalcogenides, and their tunable ion-intercalation chemistry makes them of considerable interest for electrochemical applications. However, the conventional synthesis of high-purity Chevrel phases generally relies on prolonged high-temperature sintering, which is unfavorable for rapid materials screening and compositional regulation. Herein, we propose a Joule-heating strategy that enables the rapid synthesis of Cu-intercalated Mo_6_S_8_ Chevrel phases with a holding time of 30 s at 1500 °C. Among the prepared Cu_*x*_Mo_6_S_8_ samples, Cu_2.76_Mo_6_S_8_ exhibits a high phase purity of 99.6% and a relatively uniform Cu distribution within the examined particles. Hydrogen evolution reaction (HER) measurements show that Cu_2.76_Mo_6_S_8_ displays a nearly pH-symmetric electrocatalytic response, requiring overpotentials of 403 and 400 mV to reach a current density of 10 mA cm^−2^ in 0.5 M H_2_SO_4_ and 1.0 M KOH, respectively. The corresponding Tafel slopes are also comparable in acidic and alkaline electrolytes, indicating relatively balanced HER kinetics under different pH conditions. Further electrochemical cycling tests reveal electrolyte-dependent structural reconstruction: enhanced HER activity in acidic media is accompanied by structural changes consistent with partial Cu deintercalation, whereas moderate catalytic degradation in alkaline media is accompanied by more complex evolution of Cu species. XRD and XPS characterizations indicate that the Mo_6_S_8_ cluster framework is largely retained after cycling, while the Cu species undergo electrolyte-dependent evolution. This work provides an effective route for the rapid preparation of high-purity Chevrel phases and demonstrates that Cu-intercalated Mo_6_S_8_ can serve as a model system for investigating pH-dependent HER behavior and electrochemical reconstruction mechanisms.

## Abstract 2

Joule heating yields high-purity Cu_*x*_Mo_6_S_8_ Chevrel phases with a 30 s hold. Cu_2.76_Mo_6_S_8_ shows nearly identical HER overpotentials in acidic and alkaline media, alongside electrolyte-dependent reconstruction.

## Graphical abstract

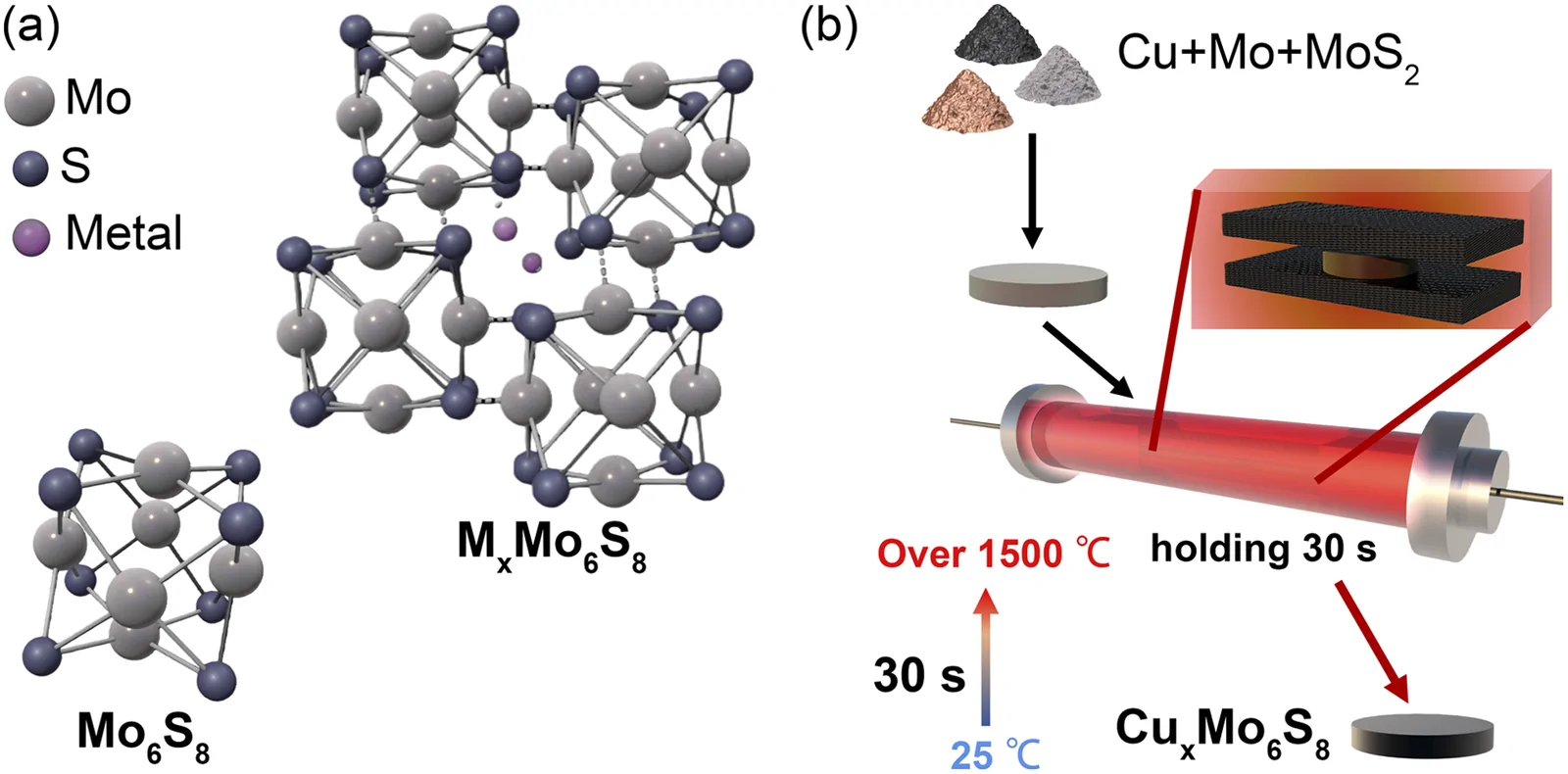

## Introduction

Hydrogen is widely regarded as an important clean energy carrier because of its high gravimetric energy density and environmentally benign combustion product.^1–3^ Among various hydrogen-production technologies, electrocatalytic water splitting powered by renewable energy offers a scalable and potentially sustainable route for hydrogen production.^2–5^ The hydrogen evolution reaction (HER), as the cathodic half-reaction of water electrolysis, therefore plays a central role in determining the energy efficiency and practical viability of electrolytic hydrogen production.

Pt-group noble metals remain the benchmark HER catalysts because of their favorable hydrogen adsorption energetics and fast reaction kinetics.^6–10^ However, their scarcity and high cost restrict large-scale deployment.^10,11^ Considerable effort has therefore been devoted to developing non-noble-metal catalysts that can operate efficiently and stably under different electrolyte conditions. In addition to activity, pH compatibility is also important. Although many catalysts exhibit high HER activity in acidic media, their activity is often lower in alkaline electrolytes, where the formation of adsorbed hydrogen involves water dissociation, which can impose an additional kinetic barrier.^7,12^ Even Pt exhibits much slower HER kinetics in alkaline media than in acidic media.^13,14^ Evaluating the HER behavior of the same catalyst in both acidic and alkaline electrolytes provides a basis for examining how the electrolyte environment influences catalytic activity and stability.^6,15^

Chevrel-phase compounds represent a family of cluster-based transition-metal chalcogenides with the general formula M_*x*_Mo_6_T_8_, where M is an intercalated metal and T is S, Se, or Te.^16–18^ Their interconnected Mo_6_T_8_ cluster units form a three-dimensional framework that can accommodate intercalated metal species.^17,19^ Metal intercalation provides a means of modulating their electronic structure and HER activity.^20^ Chevrel phases have consequently been investigated as HER electrocatalysts in both acidic and alkaline electrolytes.^21–23^ For example, hollow Chevrel-type NiMo_3_T_4_ nanoparticles and interfacially engineered CuMo_6_S_8_/Cu electrodes have demonstrated the potential of Mo–S cluster-based materials for hydrogen evolution.^22,23^ Nevertheless, most reported Chevrel-phase electrocatalysts have been investigated mainly under alkaline conditions, while their behavior in acidic media and the associated pH-dependent structural evolution remain less understood.

Another challenge lies in synthesis. Conventional Chevrel-phase synthesis usually relies on long-duration high-temperature solid-state reactions, often requiring tens of hours or even several days to obtain high-purity products.^19,24–26^ Such time-consuming processes not only increase energy consumption but also impede rapid composition optimization and electrochemical screening. Recent studies have further explored Joule heating as a rapid synthesis strategy for nanomaterials, highlighting its growing relevance to materials preparation.^27–30^ Ninawe *et al.*^31^ demonstrated the rapid synthesis of Cu_2_Mo_6_S_8_ by flash-Joule heating, highlighting the potential of electrically driven thermal processing for accelerating Chevrel-phase formation. Further exploration of rapid synthesis routes and the electrochemical behavior of the resulting materials is therefore valuable for advancing Chevrel-phase electrocatalysts.

Here, we employ Joule heating to prepare a series of Cu_*x*_Mo_6_S_8_ samples and investigate their composition-dependent HER responses in acidic and alkaline electrolytes, together with the phase and compositional changes observed after electrochemical testing. By varying the Cu content, a series of Cu_*x*_Mo_6_S_8_ compounds are obtained, among which Cu_2.76_Mo_6_S_8_ exhibits the highest phase purity of 99.6%. Cu_2.76_Mo_6_S_8_ shows a nearly identical overpotential at 10 mA cm^−2^ in acidic and alkaline media, together with comparable Tafel slopes, indicating a distinctive pH-symmetric HER behavior. Moreover, cycling tests combined with XRD and XPS analyses reveal that Cu species undergo electrolyte-dependent deintercalation or reaction, whereas the Mo_6_S_8_ cluster framework is largely retained. These findings provide insight into the electrochemical behavior of Cu-intercalated Chevrel phases and demonstrate Joule heating as an efficient strategy for their rapid preparation.

## Experimental

### Materials

All the raw materials, including Cu powder (2 N, 99.7%), Mo powder (2 N, 99.0%) and MoS_2_ powder (98%) were purchased from Sinopharm Chemical Reagent Co., Ltd (Shanghai, China).

### Methods

#### Synthesis

Three Cu_*x*_Mo_6_S_8_ materials were synthesized *via* a high-temperature thermal shock strategy. Specifically, Cu, Mo, and MoS_2_ powders were mixed in molar ratios of 2 : 2 : 4, 4 : 2 : 4, and 6 : 2 : 4, respectively, and thoroughly ground to ensure homogeneity. The resulting mixtures were then pressed into cylindrical pellets (diameter: *φ*10 mm) under a pressure of 10 MPa for 5 minutes. Each pellet was sandwiched between carbon strips and subjected to rapid heating to 1500 °C within 30 seconds under an argon atmosphere, held at this temperature for 30 seconds, and subsequently quenched to room temperature. The final products were denoted as Cu_2_Mo_6_S_8_, Cu_2.76_Mo_6_S_8_, and Cu_3.66_Mo_6_S_8_, corresponding to the three initial stoichiometries.

The catalyst ink was prepared by ultrasonically dispersing 5 mg of catalyst powder in a mixture of 50 µL of Nafion solution and 950 µL of isopropanol/water solvent, yielding a catalyst concentration of 5 mg mL^−1^. Subsequently, 1 µL of the ink was deposited onto a glassy carbon electrode (3 mm in diameter; geometric area: 0.0707 cm^2^), corresponding to a catalyst mass of 5 µg and a mass loading of 0.0707 mg cm^−2^.

#### Characterization

XRD patterns were measured by an X-ray diffractometer (PANalytical, X'Pert3Powder), SEM images was acquired by Scanning Electron Microscope (Hitachi, SU6600), TEM images were acquired by Transmission Electron Microscope (JEOL, JEM-2100Plus), XPS spectra were recorded using an XPS spectrometer (Thermo Fisher Scientific, ESCALAB 250 Xi).

#### Electrochemical measurements

Electrochemical measurements for the hydrogen evolution reaction (HER) were carried out in both 0.5 M H_2_SO_4_ (acidic) and 1.0 M KOH (alkaline) using a DH7000 electrochemical workstation with a standard three-electrode configuration. Prior to polarization measurements, 30 cyclic voltammetry (CV) cycles were performed in the potential window of −0.8 to 0 V *vs.* RHE at a scan rate of 100 mV s^−1^ to stabilize the electrode surface. Linear sweep voltammetry (LSV) was then recorded at a slow scan rate of 2 mV s^−1^ over −0.8 to 0 V *vs.* RHE for HER. All LSV data were iR-corrected by compensating 85% of the solution resistance (determined from EIS), and Tafel plots were derived from the corrected LSV curves. Electrochemical impedance spectroscopy (EIS) was conducted at the overpotentials corresponding to 10 mA cm^−2^ for HER in each electrolyte, using a sinusoidal voltage perturbation of 5 mV over a frequency range of 10^5^ to 10^−1^ Hz. Cycling stability was assessed by comparing the HER polarization curves before and after 1000 cyclic voltammetry (CV) cycles, including the overpotentials at 10 mA cm^−2^. Separately, long-term HER stability was evaluated by 100 h potentiostatic chronoamperometry at room temperature in both acidic and alkaline media.

## Results and discussion

### Ultrafast Joule-heating synthesis of Cu_*x*_Mo_6_S_8_ Chevrel phases

The crystal structure of Chevrel-phase compounds is built from Mo_6_S_8_ cluster units, into which metal atoms can be intercalated to form ternary M_*x*_Mo_6_S_8_ phases. A schematic illustration of the Mo_6_S_8_ cluster and Cu-intercalated Cu_*x*_Mo_6_S_8_ structure is shown in Fig. 1a. In this work, Cu_*x*_Mo_6_S_8_ was synthesized by a Joule-heating route, as illustrated in Fig. 1b. Briefly, Cu, MoS_2_ and Mo powders with designed molar ratios were thoroughly mixed and pressed into pellets. The pellets were sandwiched between two Joule-heating carbon strips and rapidly heated to 1500 °C under an Ar atmosphere, held for 30 s, and then quenched to room temperature.

**Fig. 1:**
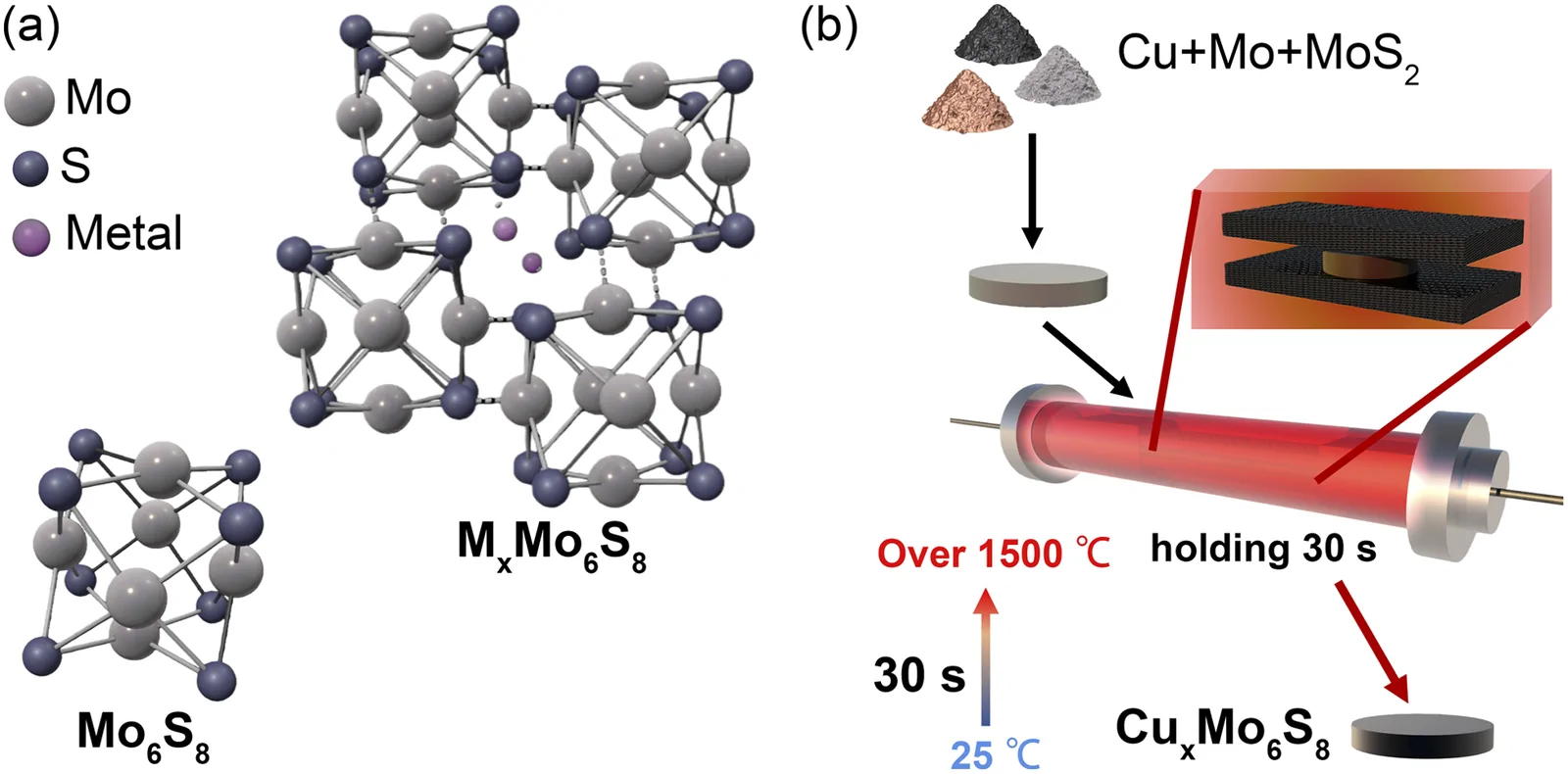
Schematic illustration of the Chevrel-phase structure and Joule-heating synthesis. (a) Mo_6_S_8_ cluster and Cu-intercalated Cu_*x*_Mo_6_S_8_ Chevrel phase. (b) Schematic of the Joule-heating synthesis process for Cu_*x*_Mo_6_S_8_.

This rapid heating process enables the direct formation of Cu_*x*_Mo_6_S_8_ Chevrel phases within an exceptionally short reaction time. Fig. 2a–c show the Rietveld-refined powder X-ray diffraction patterns of Cu_2_Mo_6_S_8_, Cu_2.76_Mo_6_S_8_ and Cu_3.66_Mo_6_S_8_, respectively. As the Cu content in the precursor increases, the Cu content in the final Cu_*x*_Mo_6_S_8_ product also increases. The phase purities of Cu_2_Mo_6_S_8_, Cu_2.76_Mo_6_S_8_ and Cu_3.66_Mo_6_S_8_ are 95.4%, 99.6% and 92.1%, respectively. The main impurity in Cu_2_Mo_6_S_8_ is residual MoS_2_, whereas residual Mo is detected in Cu_2.76_Mo_6_S_8_ and Cu_3.66_Mo_6_S_8_. Among these samples, Cu_2.76_Mo_6_S_8_ shows the highest phase purity, indicating that an appropriate Cu content is beneficial for the rapid formation of high-purity Chevrel phase under Joule-heating conditions. The nominal precursor Cu content does not directly correspond to the Cu content incorporated into the final Chevrel phase. Copper-colored deposits observed on the carbon-strip surfaces after synthesis suggest that some Cu may be transferred outside the pellet through volatilization and deposition. Partial Cu volatilization has also been proposed in previous work on the self-propagating high-temperature synthesis of copper Chevrel phases.^32^

**Fig. 2:**
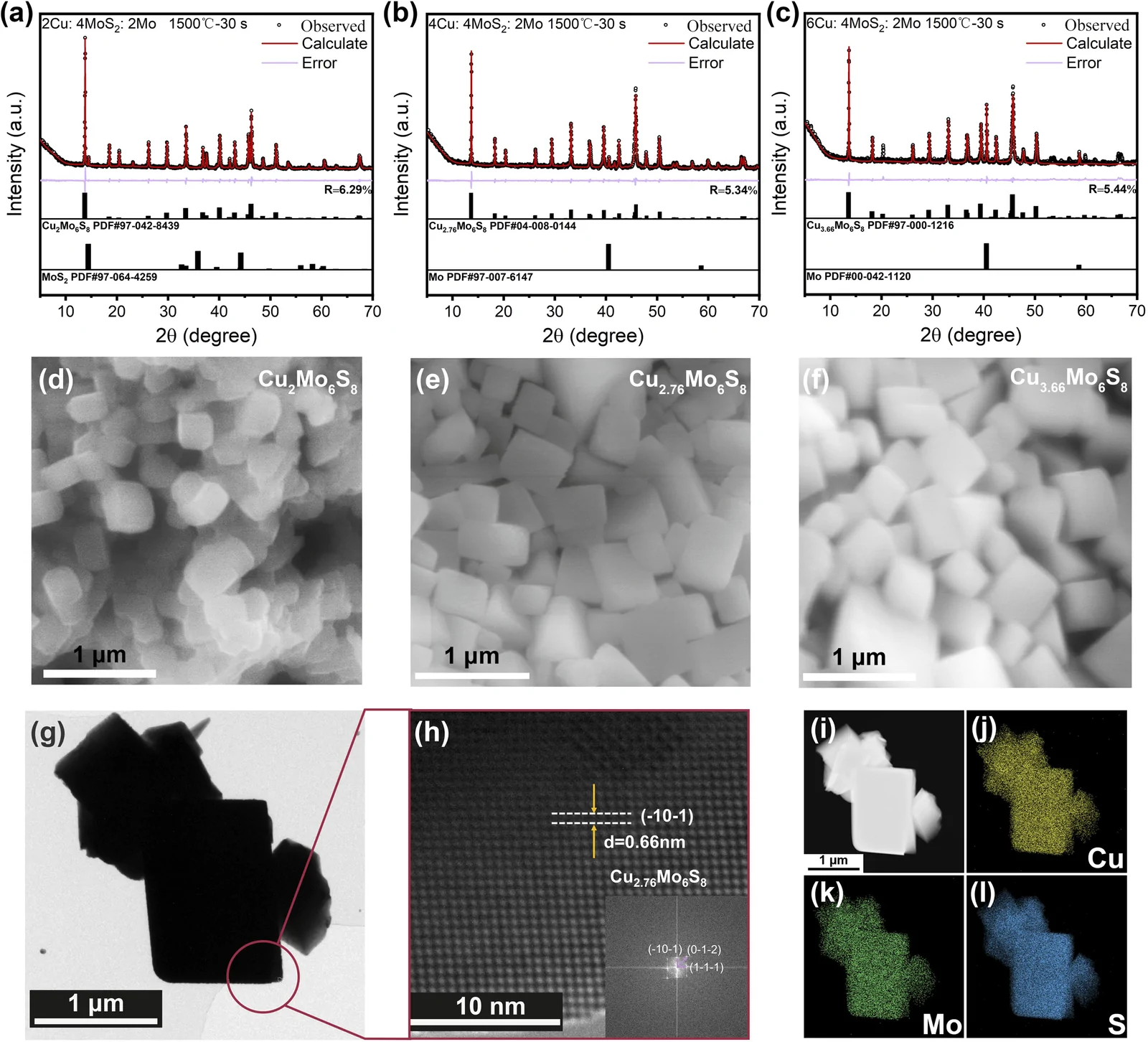
Structural and morphological characterization of Cu_*x*_Mo_6_S_8_ synthesized by Joule heating. (a–c) Rietveld refinements of the PXRD patterns of Cu_2_Mo_6_S_8_, Cu_2.76_Mo_6_S_8_ and Cu_3.66_Mo_6_S_8_. (d–f) SEM images of Cu_2_Mo_6_S_8_, Cu_2.76_Mo_6_S_8_ and Cu_3.66_Mo_6_S_8_. (g) HRTEM image of Cu_2.76_Mo_6_S_8_. (h) Enlarged HRTEM image taken from the marked region in (g); the inset shows the corresponding FFT pattern. (i–l) HAADF-STEM image and elemental mappings of Cu_2.76_Mo_6_S_8_.

The morphology and microstructure of the synthesized samples were further examined by SEM and TEM. As shown in Fig. 2d–f, Cu_2_Mo_6_S_8_, Cu_2.76_Mo_6_S_8_ and Cu_3.66_Mo_6_S_8_ all exhibit cubic-like particles with relatively uniform morphology. High-resolution TEM of Cu_2.76_Mo_6_S_8_ shows a clear lattice fringe with a spacing of 0.66 nm, which can be assigned to the (−1 0 −1) plane of Cu_2.76_Mo_6_S_8_. The corresponding fast Fourier transform pattern further confirms the crystalline nature of the Chevrel phase. HAADF-STEM imaging and the corresponding elemental maps indicate a relatively uniform Cu distribution within the examined particles. However, spectral overlap between Mo and S limits the independent assessment of their spatial distributions from these maps.

### HER behavior of Cu_*x*_Mo_6_S_8_ in alkaline electrolyte

The HER performance of Cu_2_Mo_6_S_8_, Cu_2.76_Mo_6_S_8_ and Cu_3.66_Mo_6_S_8_ was first evaluated in 1.0 M KOH. Bulk MoS_2_ and elemental Mo were tested as references because both were identified as residual phases in the synthesized samples. Fig. 3a shows the linear sweep voltammetry curves of the samples in alkaline medium, and the corresponding overpotentials at 10 mA cm^−2^ are summarized in Fig. 3b. Bulk MoS_2_ requires an overpotential of 569 mV, indicating its limited activity in the present electrode configuration. Elemental Mo requires an overpotential of 601 mV to reach 10 mA cm^−2^ in 1.0 M KOH (Fig. S2a), higher than those of all three Cu_*x*_Mo_6_S_8_ samples. In comparison, Cu_2_Mo_6_S_8_, Cu_2.76_Mo_6_S_8_ and Cu_3.66_Mo_6_S_8_ require overpotentials of 540, 400 and 452 mV, respectively. Among them, Cu_2.76_Mo_6_S_8_ exhibits the lowest overpotential, suggesting that an intermediate Cu content provides a more favorable HER response in alkaline electrolyte.

**Fig. 3:**
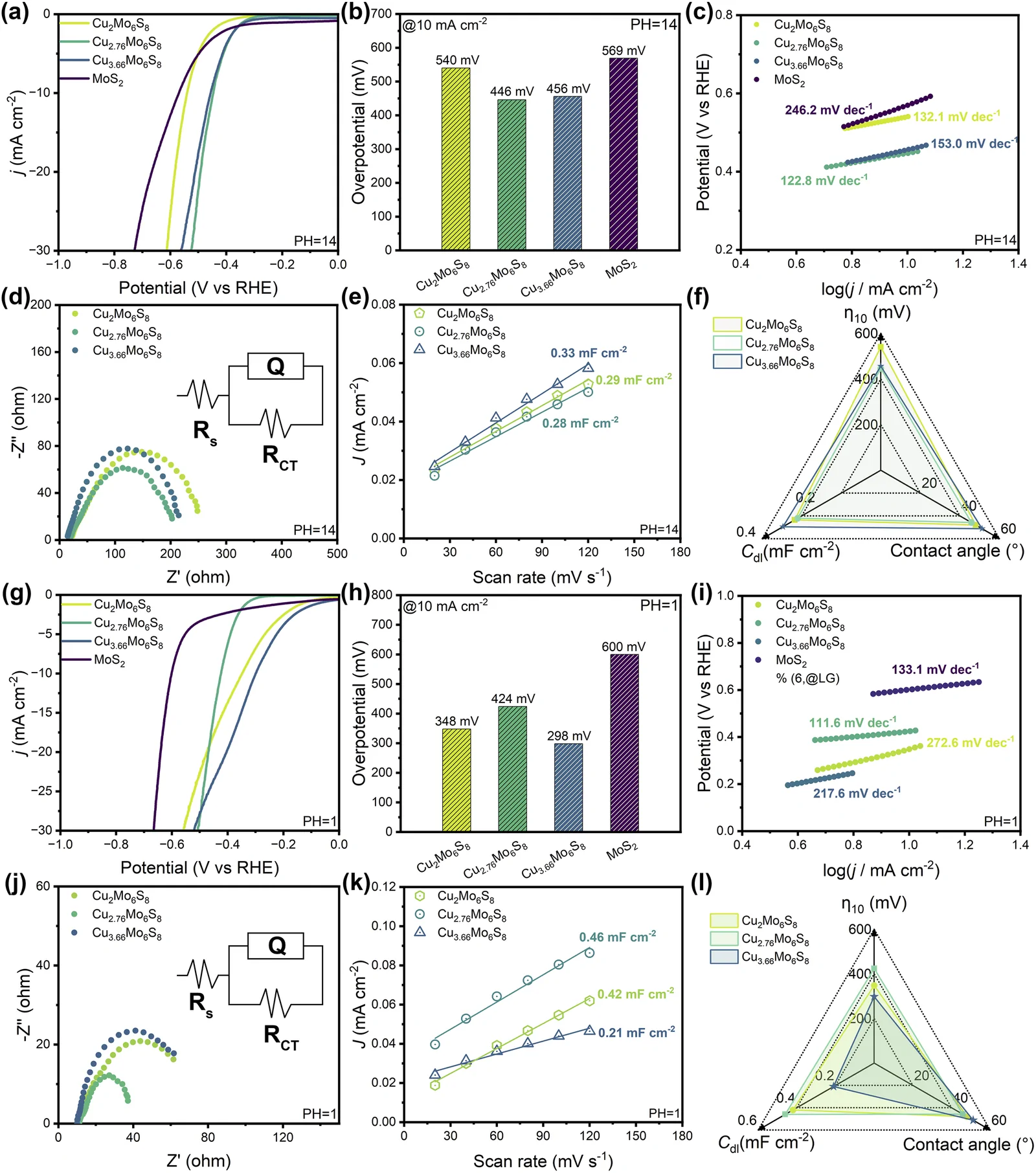
HER performance and pH-symmetric response of Cu_*x*_Mo_6_S_8_. (a) LSV curves in 1.0 M KOH. (b) Overpotentials at 10 mA cm^−2^ (*η*_10_) in 1.0 M KOH. (c) Tafel slopes in 1.0 M KOH. (d) Nyquist plots in 1.0 M KOH. (e) *C*_dl_ values obtained from CV measurements. (f) Comparison of *C*_dl_, water contact angle and *η*_10_ in 1.0 M KOH. (g) LSV curves in 0.5 M H_2_SO_4_. (h) Overpotentials at 10 mA cm^−2^ (*η*_10_) in 0.5 M H_2_SO_4_. (i) Tafel slopes in 0.5 M H_2_SO_4_. (j) Nyquist plots in 0.5 M H_2_SO_4_. (k) *C*_dl_ values obtained from CV measurements in 0.5 M H_2_SO_4_. (l) Comparison of *C*_dl_, water contact angle and *η*_10_ in 0.5 M H_2_SO_4_.

Tafel analysis was performed to evaluate the reaction kinetics. The Tafel slopes of Cu_2_Mo_6_S_8_, Cu_2.76_Mo_6_S_8_ and Cu_3.66_Mo_6_S_8_ and MoS_2_ are 132.1, 122.8, 153.0 and 246.2 mV dec^−1^, respectively (Fig. 3c). Cu_2.76_Mo_6_S_8_ shows the smallest Tafel slope among the Cu_*x*_Mo_6_S_8_ samples, indicating relatively faster HER kinetics. Electrochemical impedance spectroscopy further supports this trend. As shown in Fig. 3d, the charge-transfer resistance values of Cu_2_Mo_6_S_8_, Cu_2.76_Mo_6_S_8_ and Cu_3.66_Mo_6_S_8_ are 247.2, 213.0 and 217.8 Ω, respectively, with Cu_2.76_Mo_6_S_8_ showing the lowest resistance.

The electrochemically active surface area was estimated from the double-layer capacitance. As shown in Fig. 3e, the *C*_dl_ values of Cu_2_Mo_6_S_8_, Cu_2.76_Mo_6_S_8_ and Cu_3.66_Mo_6_S_8_ are 0.29, 0.28 and 0.33 mF cm^−2^, respectively. It could be seen in Fig. 3f the *C*_dl_ values of the three samples are comparable, suggesting that the activity difference is not simply caused by a larger electrochemically accessible surface area. Contact-angle measurements further show similar wettability for the three samples, with values of 48.0°, 45.8° and 51.2°, respectively. Therefore, the better alkaline HER performance of Cu_2.76_Mo_6_S_8_ is more likely related to Cu-intercalation-modulated intrinsic activity rather than differences in surface area or wettability.

### HER behavior of Cu_*x*_Mo_6_S_8_ in acidic electrolyte and pH-symmetric response

The HER performance of Cu_*x*_Mo_6_S_8_ was further evaluated in 0.5 M H_2_SO_4_. Fig. 3g displays the LSV curves, and the corresponding overpotentials at 10 mA cm^−2^ are summarized in Fig. 3h. Cu_2_Mo_6_S_8_, Cu_2.76_Mo_6_S_8_ and Cu_3.66_Mo_6_S_8_ require overpotentials of 348, 403 and 490 mV, respectively. Under acidic conditions, elemental Mo requires an overpotential of 552 mV at 10 mA cm^−2^ (Fig. S2b), again exceeding those of all three Cu_*x*_Mo_6_S_8_ samples. These comparisons suggest that residual Mo alone is unlikely to account for the observed HER performance of the Chevrel-phase samples. In contrast to the alkaline electrolyte, Cu_2_Mo_6_S_8_ shows the lowest overpotential in acid. However, its Tafel slope is relatively large, reaching 272.6 mV dec^−1^. The Tafel slopes of Cu_2.76_Mo_6_S_8_ and Cu_3.66_Mo_6_S_8_ are 111.6 and 217.6 mV dec^−1^, respectively (Fig. 3i), indicating that Cu_2.76_Mo_6_S_8_ maintains the most favorable reaction kinetics among the three samples in acidic medium. This is also supported by EIS results, where Cu_2.76_Mo_6_S_8_ shows the lowest charge-transfer resistance of 29.1 Ω, compared with 73.2 Ω for Cu_2_Mo_6_S_8_ and 70.9 Ω for Cu_3.66_Mo_6_S_8_ (Fig. 3j).

The electrochemically active surface area in acidic electrolyte was also estimated from the double-layer capacitance. As shown in Fig. 3k, Cu_3.66_Mo_6_S_8_ exhibits a much smaller *C*_dl_ value than Cu_2_Mo_6_S_8_ and Cu_2.76_Mo_6_S_8_, indicating a lower electrochemically accessible surface area. To further correlate surface properties with HER activity, the *C*_dl_ values, water contact angles (Fig. S3) and overpotentials at 10 mA cm^−2^ in acidic electrolyte are compared in Fig. 3l. Although Cu_2_Mo_6_S_8_ shows the lowest overpotential in acidic medium, Cu_2.76_Mo_6_S_8_ exhibits the smallest Tafel slope and charge-transfer resistance, suggesting more favorable reaction kinetics. These results indicate that the acidic HER behavior of Cu_*x*_Mo_6_S_8_ is not governed solely by electrochemically active surface area or wettability, but is strongly affected by Cu-intercalation-regulated intrinsic charge-transfer kinetics.

A notable feature of Cu_2.76_Mo_6_S_8_ is its balanced HER response in acidic and alkaline electrolytes. Although its absolute overpotential is not lower than state-of-the-art HER catalysts, the overpotential required to reach 10 mA cm^−2^ is nearly identical in acid and alkali, with values of 403 and 400 mV, respectively. This corresponds to a pH asymmetry of only 3 mV, much smaller than those of Cu_2_Mo_6_S_8_ and Cu_3.66_Mo_6_S_8_. A similar trend is observed for the Tafel slope: the difference between acidic and alkaline media is only 11.2 mV dec^−1^ for Cu_2.76_Mo_6_S_8_, whereas Cu_2_Mo_6_S_8_ and Cu_3.66_Mo_6_S_8_ show much larger differences.

Among the samples examined, Cu_2.76_Mo_6_S_8_ exhibits the smallest differences in overpotential at 10 mA cm^−2^ and Tafel slope between acidic and alkaline electrolytes. This establishes a correlation between the initial sample composition and the measured HER response, but does not identify the surface state responsible for this behavior. Whether samples with different initial Cu contents evolve toward a common composition and catalytic activity during cycling remains unresolved. The present measurements also do not isolate the respective contributions of Cu deintercalation, accompanying surface changes, secondary phases, and Cu species in the electrolyte.

To further evaluate long-term operational stability, 100 h chronoamperometric measurements were performed in 0.5 M H_2_SO_4_ and 1.0 M KOH (Fig. S4). The cathodic current magnitude increased during acidic operation, whereas it decreased to a near-zero level after approximately 48 h in alkaline electrolyte, indicating limited operational stability under alkaline conditions.

### Electrochemical reconstruction and stability of Cu_2.76_Mo_6_S_8_

To examine the phase evolution associated with the electrolyte-dependent HER behavior, XRD measurements were performed after 1000 CV cycles. After acidic cycling, the diffraction pattern exhibits features consistent with a CuMo_6_S_8_-like phase (Fig. S5a), suggesting that a Chevrel-type crystalline framework persists despite changes in Cu content. After alkaline cycling, reflections attributable to a copper sulfide phase are also observed alongside those of the Chevrel-type phase (Fig. S5b), indicating a more complex phase evolution. These observations are consistent with partial Cu deintercalation in acidic electrolyte and additional phase segregation or reconstruction in alkaline electrolyte, although the diffraction patterns alone do not establish the detailed transformation pathways.

The observation of a CuMo_6_S_8_-like phase after cycling in both electrolytes does not imply identical catalytic surfaces, particularly because additional copper sulfide reflections are observed after alkaline cycling. The opposite activity trends therefore cannot be explained by the common crystalline phase alone. In acidic electrolyte, the improved HER response may involve changes in the local environment of Mo–S sites associated with Cu depletion and/or an increase in electrochemically accessible surface area during reconstruction. However, the present data do not distinguish these contributions, and the activity enhancement cannot be assigned solely to an increase in intrinsic activity or to the formation of a CuMo_6_S_8_-like phase.

The Cu_2.76_Mo_6_S_8_ composition was determined by Rietveld refinement of the XRD data. The ICP results are used to assess relative compositional changes before and after electrolysis rather than to establish the exact stoichiometry of the Chevrel phase. ICP analysis revealed distinct compositional changes after 100 h of potentiostatic HER testing in acidic and alkaline electrolytes (Table S1). In acidic electrolyte, the Cu content decreased from 23.07 to 16.96 wt%, accompanied by a decrease in the Cu/Mo atomic ratio from 0.689 to 0.454. The S/Mo ratio remained comparatively unchanged, decreasing slightly from 1.260 to 1.223. This preferential Cu depletion, together with the retention of a CuMo_6_S_8_-like phase after 1000 CV cycles (Fig. S5a), is consistent with partial Cu removal while preserving a Chevrel-type crystalline framework.

In alkaline electrolyte, the Cu/Mo atomic ratio changed only modestly, from 0.689 to 0.725, whereas the S/Mo ratio decreased markedly from 1.260 to 0.893. The sulfur depletion observed after prolonged electrolysis and the additional copper sulfide phase detected after CV cycling (Fig. S5b) suggest more complex reconstruction, potentially involving partial decomposition and redistribution of Cu species. These contrasting trends indicate that the electrolyte environment influences both the elemental composition and phase evolution of the catalyst. XPS analysis was further conducted to probe the chemical-state evolution of Cu_2.76_Mo_6_S_8_ after electrochemical cycling. The Cu XPS spectra (Fig. S6) show clear changes after both acidic and alkaline treatment, confirming that the Cu species are chemically labile during HER operation. In acidic electrolyte, the spectral evolution is consistent with partial Cu deintercalation from the Chevrel framework, while in alkaline electrolyte, the changes suggest a more complex transformation of Cu species, possibly involving the formation of copper sulfide/oxide/hydroxide-related species.

The high-resolution Mo 3d XPS spectra are shown in Fig. 4c. After cycling in 0.5 M H_2_SO_4_, the overall spectral profile remains broadly similar to that of the as-synthesized sample, while the principal Mo 3d features shift toward lower binding energies. Together with the retention of a Chevrel-phase-related crystalline structure in the post-cycling XRD pattern, these observations support substantial preservation of the framework under acidic cycling conditions. The binding-energy shift suggests changes in the electronic environment of Mo accompanying Cu depletion. In contrast, cycling in 1 M KOH produces pronounced changes in the Mo 3d spectral profile, suggesting more substantial changes in the surface chemical environments of Mo, potentially involving altered Mo–S coordination and the formation of additional Mo-containing surface species. These spectral changes are consistent with the electrolyte-dependent phase evolution observed by XRD and complement the contrasting current responses recorded during the separate 100 h potentiostatic tests.

**Fig. 4:**
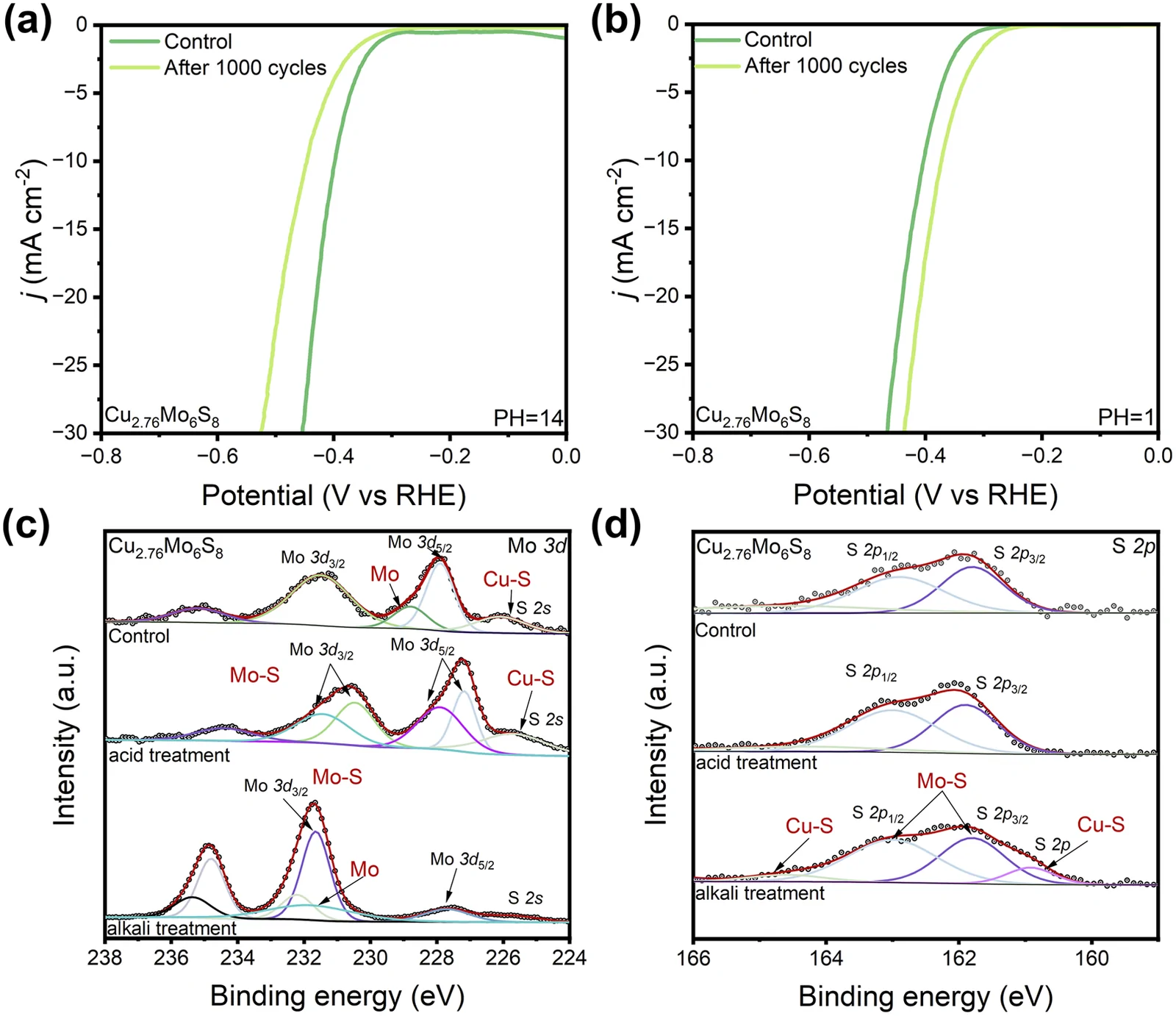
Electrochemical reconstruction of Cu_2.76_Mo_6_S_8_ after HER cycling. (a) LSV curves before and after 1000 CV cycles in 1.0 M KOH. (b) LSV curves before and after 1000 CV cycles in 0.5 M H_2_SO_4_. (c and d) XPS patterns of pristine, acid-cycled and alkali-cycled Cu_2.76_Mo_6_S_8_. (c) High-resolution Mo 3d XPS spectra. (d) High-resolution S 2p XPS spectra.

The S 2p spectra show comparatively small changes after acidic and alkaline cycling (Fig. 4d), suggesting that the dominant sulfur chemical environments detected by XPS remain broadly similar.

Overall, the XRD and XPS results suggest electrolyte-dependent evolution of Cu species, while the Mo_6_S_8_ framework is largely retained. The improved HER response after acidic cycling is accompanied by structural changes consistent with partial Cu deintercalation and the formation of a Cu-depleted, CuMo_6_S_8_-like phase. This correlation suggests that Cu-related reconstruction may contribute to the activation process. However, without a directly synthesized, high-purity CuMo_6_S_8_ reference tested under comparable conditions, the activity enhancement cannot be attributed solely to this phase evolution. The present measurements do not distinguish the effects of Cu deintercalation from those of accompanying changes in surface composition, local structure, and interfacial properties. In alkaline electrolyte, the activity loss is accompanied by the formation of additional Cu-containing phases, although their individual contributions remain unresolved.

## Conclusions

In summary, we developed an ultrafast Joule-heating method for synthesizing Cu-intercalated Mo_6_S_8_ Chevrel phases with a holding time of 30 s at 1500 °C. By adjusting the Cu content, Cu_*x*_Mo_6_S_8_ samples with different compositions were obtained, among which Cu_2.76_Mo_6_S_8_ exhibited the highest phase purity of 99.6% and a relatively uniform Cu distribution within the examined particles. Electrochemical measurements revealed that Cu_2.76_Mo_6_S_8_ shows a distinctive pH-symmetric HER response, with overpotentials of 403 and 400 mV at 10 mA cm^−2^ in 0.5 M H_2_SO_4_ and 1.0 M KOH, respectively. The small differences in both overpotential and Tafel slope between acidic and alkaline electrolytes suggest that Cu intercalation can regulate the HER response of Mo_6_S_8_-based Chevrel phases across different pH environments. Cycling tests further revealed electrolyte-dependent reconstruction: acidic cycling produces an improved HER response accompanied by structural changes consistent with partial Cu deintercalation, whereas alkaline cycling produces moderate activity degradation accompanied by more complex evolution of Cu species. XRD and XPS analyses indicate that the Mo_6_S_8_ cluster framework remains largely stable during cycling, while Cu species are primarily responsible for the observed reconstruction behavior. This work demonstrates Joule heating as a rapid route for preparing high-purity Chevrel phases and provides insight into the pH-dependent electrochemical behavior of Cu-intercalated Mo_6_S_8_ materials.

## Conflicts of interest

There are no conflicts to declare.

## Supplementary Material

RA-016-D6RA05873F-s001

## Data Availability

The data supporting this article have been included in the supplementary information (SI). The content of the SI includes experimental section, figures and tables. Supplementary information: additional SEM and TEM images, XPS and XRD characterization data, and hydrogen evolution reaction (HER) measurement data. See DOI: https://doi.org/10.1039/d6ra05873f.
