# Supplementary material for: Thirty-second Joule-heating synthesis of Cu-intercalated Mo6S8 Chevrel phases with pH-symmetric hydrogen evolution behavior
Source: RSC Adv. 2026 Oct 19;16(48):64320–6. doi: 10.1039/d6ra05873f (PMC13678672; doi:10.1039/d6ra05873f)
Supplement: Supplementary file 1 [file RA-016-D6RA05873F-s001.pdf]

Supplementary Information (SI) for RSC Advances.
This journal is © The Royal Society of Chemistry

Supplementary Material

Thirty-second Joule-heating synthesis of Cu-intercalated Mo_6S_8 Chevrel phases with pH-symmetric hydrogen evolution behavior

Zongchen Jiang †[a,b], Wenqi Li†[c], Zhenyu Chen*[a], Shengnan Zhang*[a], Botao Shao[a], Fan Yang*[c], Ping Hu*[c], Chengshan Li[a], Pingxiang Zhang[a]

[a] Superconducting Materials Research Center, Northwest Institute for Non-ferrous Metal Research, Xi'an, 710016, China.

[b] School of materials science and Engineering, Northeastern University, Shenyang, 110004, China

[c] School of Metallurgy Engineering, Xi'an International Science and Technology Cooperation Base for Manufacturing of Special Powder and Powder Metallurgy, Xi'an University of Architecture and Technology, Xi'an 710055, China

† Zongchen Jiang and Wenqi Li contributed equally to this work.

Figures

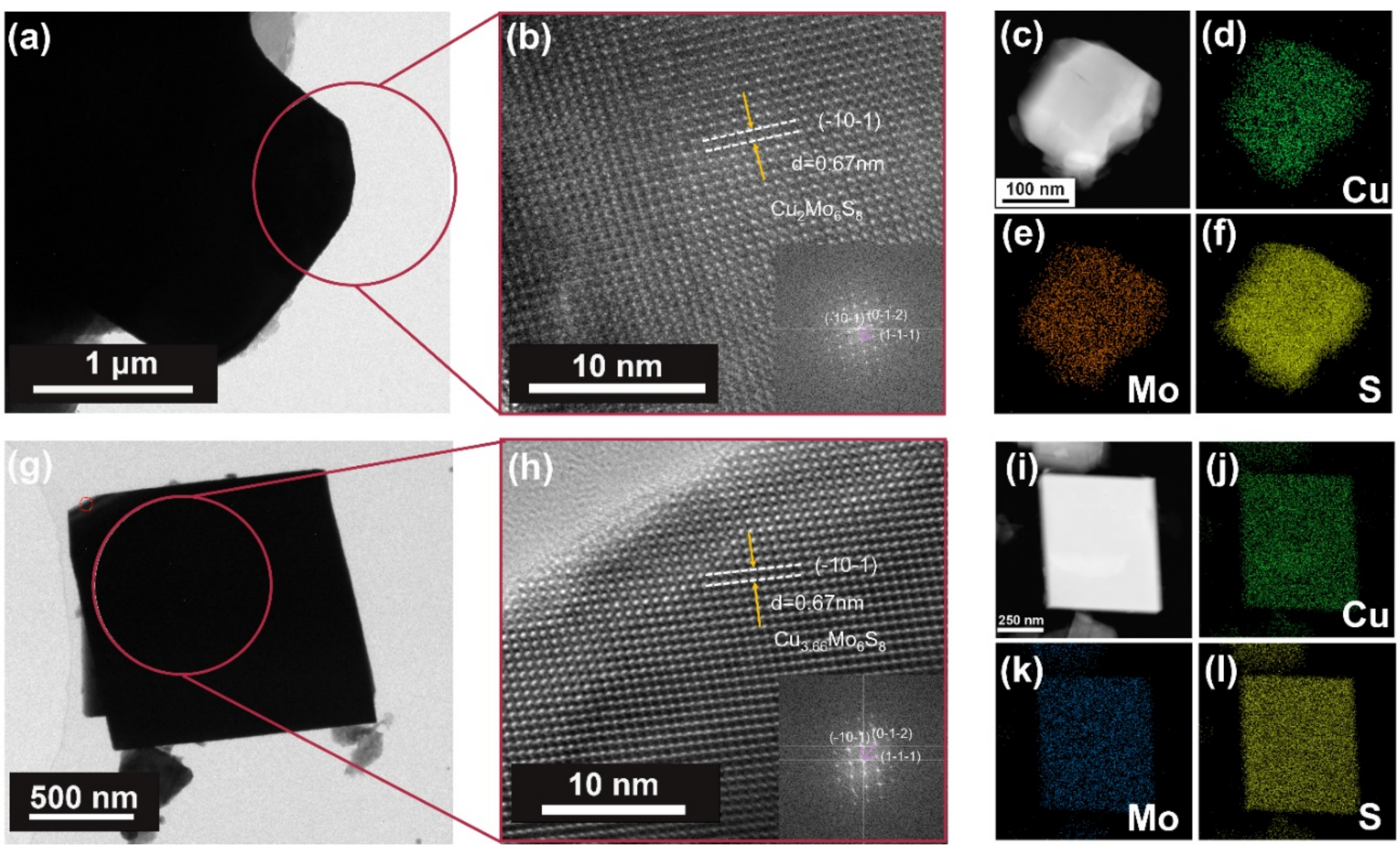

Fig. S1 Electron microscopy images and elemental maps of (a–f) $Cu_2Mo_6S_8$ and (g–l) $Cu_{3.66}Mo_6S_8$. (a, g) TEM images; (b, h) HRTEM images of the marked regions in (a, g), respectively, with the corresponding FFT patterns shown as insets; (c, i) STEM images and corresponding elemental maps of Cu (d, j), Mo (e, k), and S (f, l).

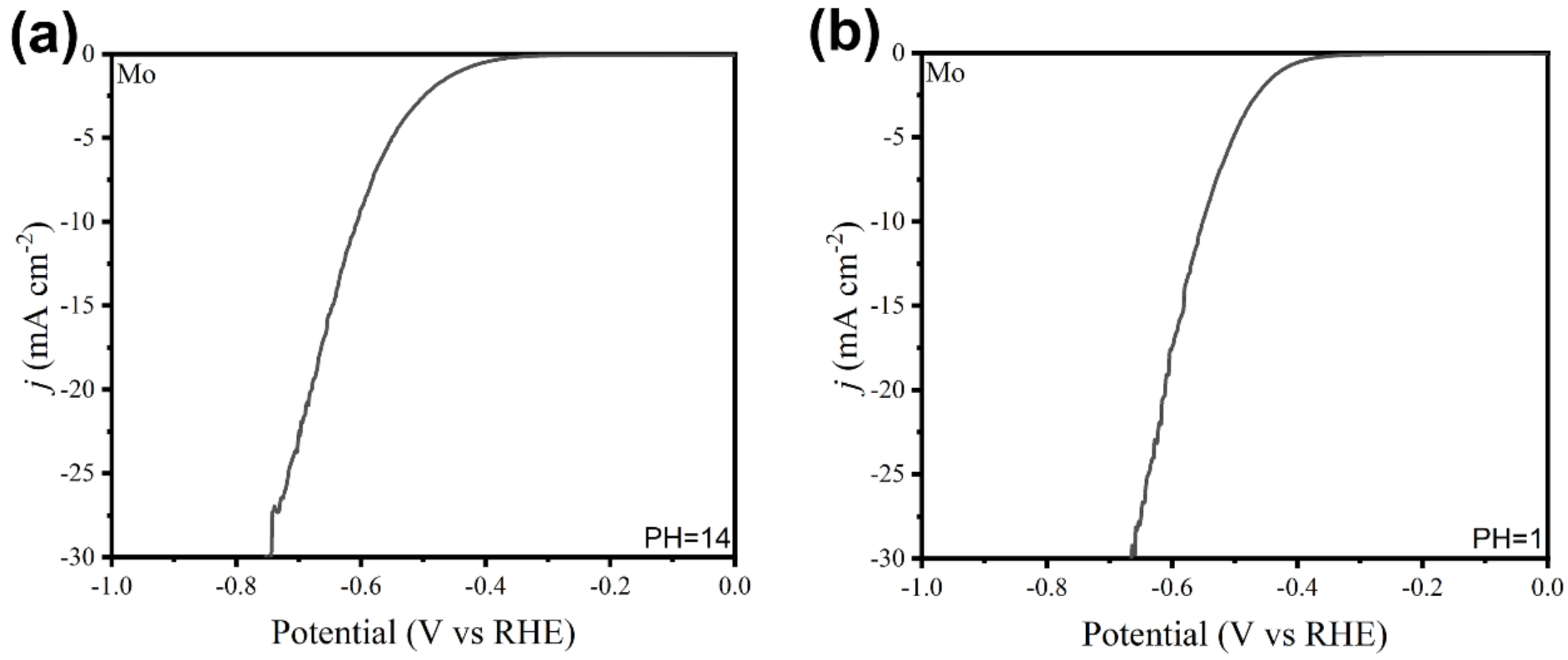

Fig. S2 LSV curves of elemental Mo in (a) 0.5 M H_2SO_4 and (b) 1.0 M KOH.

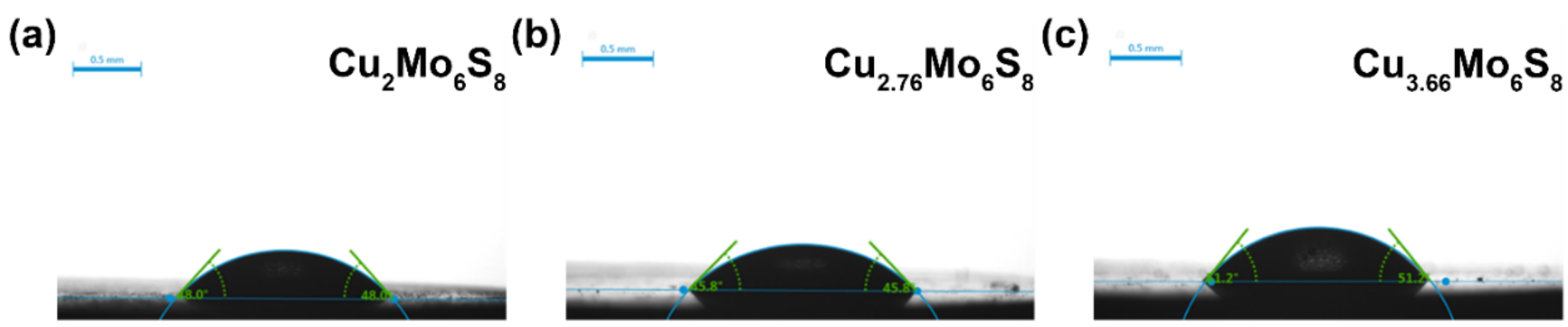

Fig. S3 Water contact angle of $Cu_2Mo_6S_8$, $Cu_{2.76}Mo_6S_8$ and $Cu_{3.66}Mo_6S_8$.

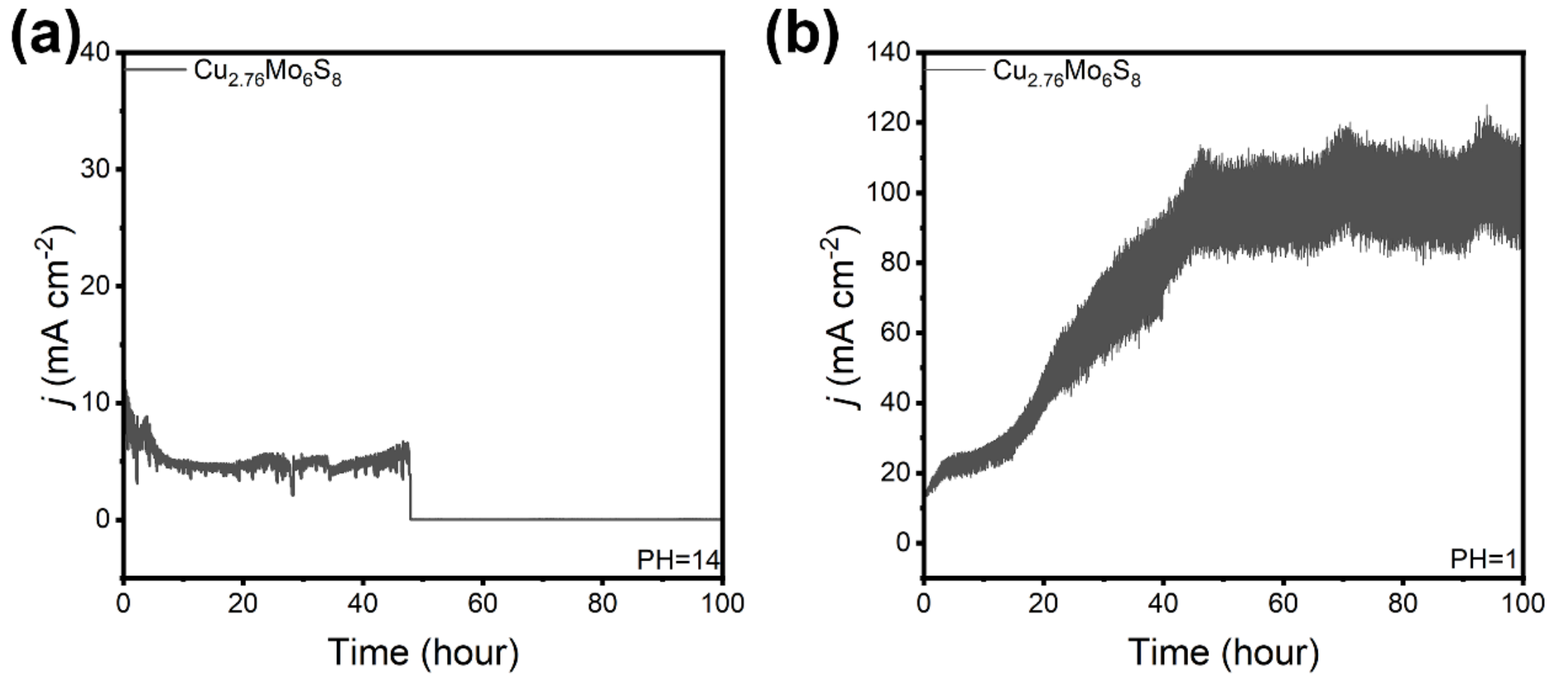

Fig. S4 Chronoamperometric curves of $Cu_{2.76}Mo_6S_8$ recorded under potentiostatic conditions for 100 h in (a) 0.5 M H_2SO_4 and (b) 1.0 M KOH.

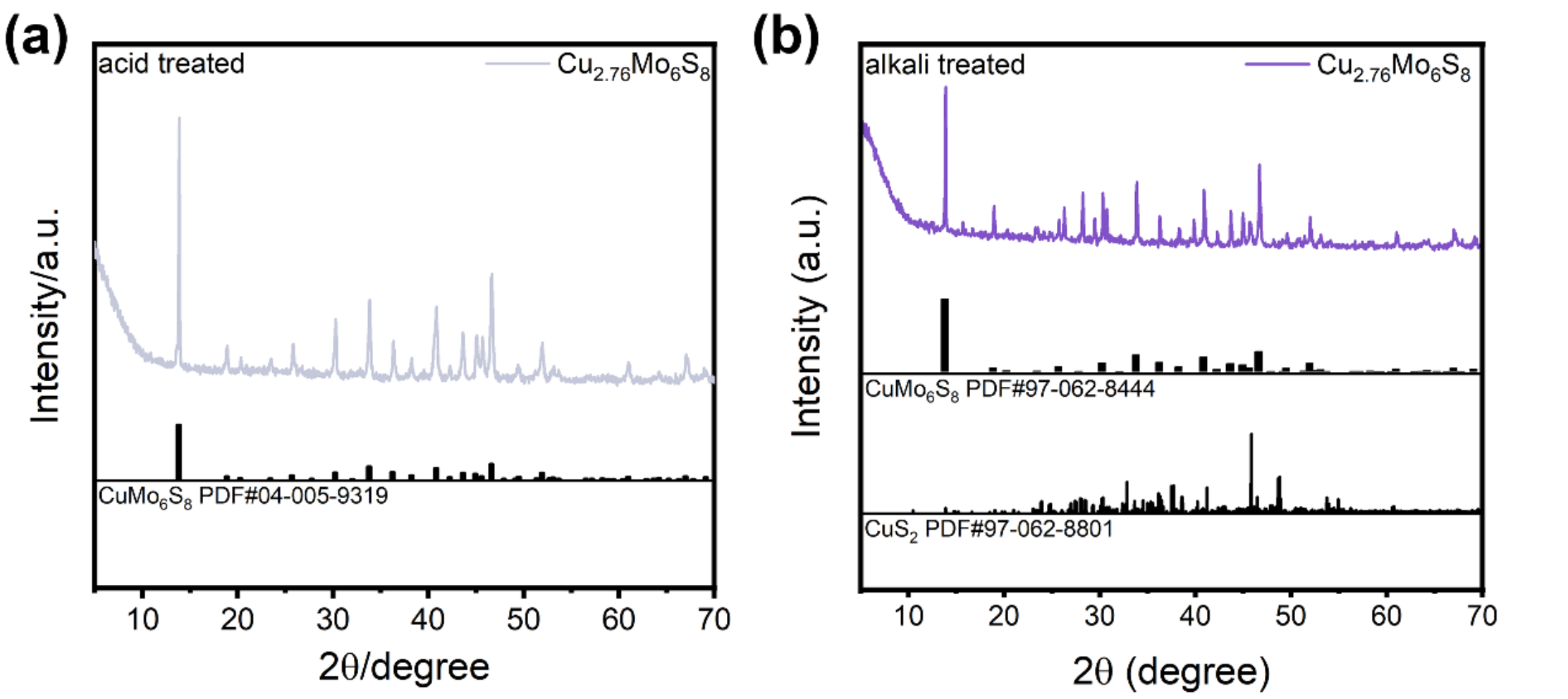

Fig. S5 XRD patterns of $Cu_{2.76}Mo_6S_8$ samples after CV cycling in (a) 0.5 M H_2SO_4 and (b) 1.0 M KOH.

\

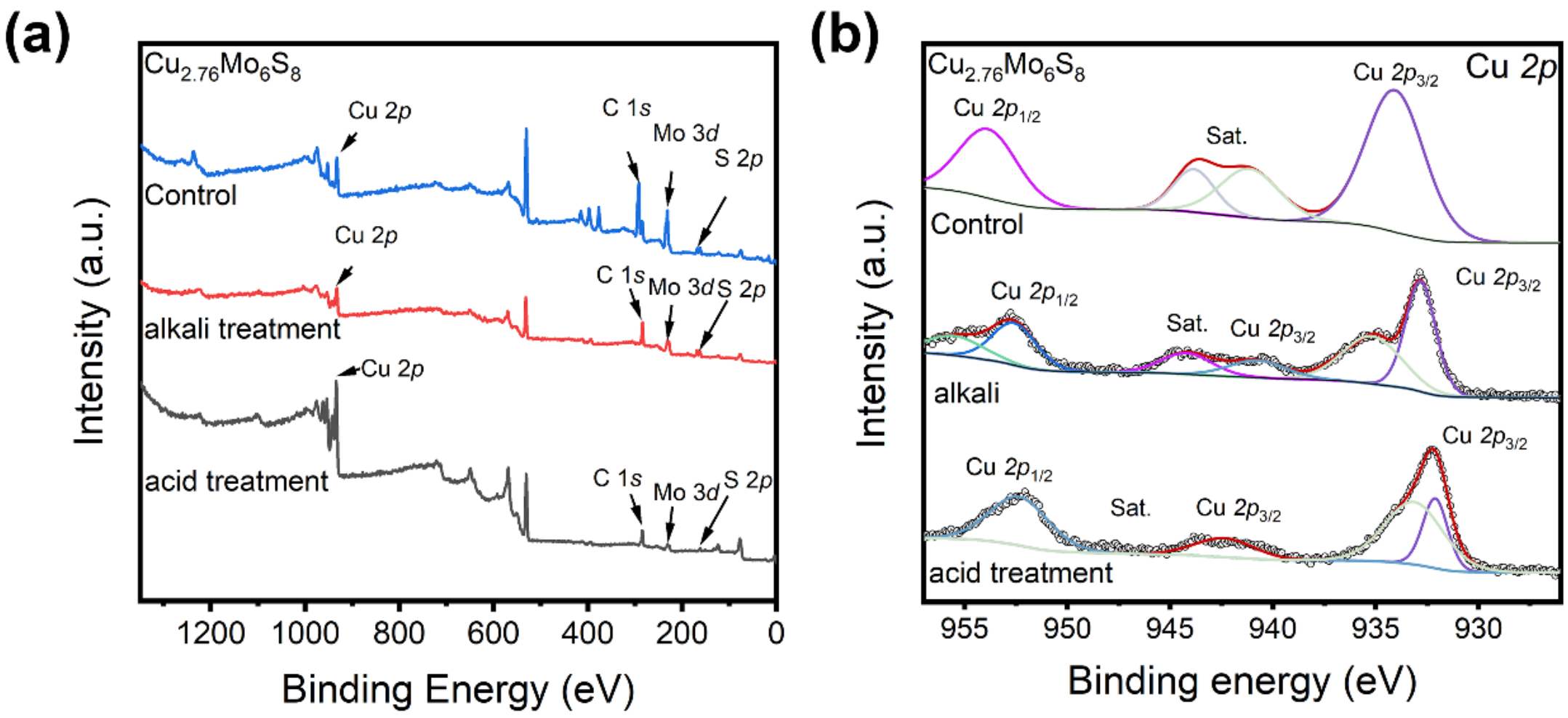

Fig. S6 (a) XPS survey spectrum of $Cu_{2.76}Mo_6S_8$; (b) High-resolution XPS spectra of $Cu_{2.76}Mo_6S_8$ (control sample, acid treated and alkali treated sample) for Mo *3d* and S *2p*.

Table S1. Elemental atomic ratios determined by ICP analysis of as-synthesized $Cu_{2.76}Mo_6S_8$ and solids recovered after separate 100 h potentiostatic HER tests in acidic and alkaline electrolytes.

Sample	Cu/Mo atomic ratio	S/Mo atomic ratio
As-synthesized	0.689	1.260
After 100 h in 0.5 M H₂SO₄	0.454	1.223
After 100 h in 1.0 M KOH	0.725	0.893
